# A Rare Case of Extensive Eggshell Intestinal Wall Peritoneal Calcification in a Long-Term Continuous Peritoneal Dialysis Patient

**DOI:** 10.1155/2022/2104120

**Published:** 2022-09-26

**Authors:** Erasmia Sampani, Chrysostomos Dimitriadis, Efstratios Kasimatis, Evangelos Memmos, Aikaterini Papagianni

**Affiliations:** Department of Nephrology, School of Medicine, Aristotle University of Thessaloniki, General Hospital “Hippokratio”, Thessaloniki, Greece

## Abstract

Encapsulating peritoneal sclerosis (EPS) is a rare but rather serious complication of long-term peritoneal dialysis. The etiology of EPS is multifactorial, with long-term peritoneal dialysis, multiple peritonitis episodes, and uncontrolled hyperparathyroidism considered to be major risk factors for this often life-threatening condition. We report a case of a 55-year-old female patient with Down syndrome and end-stage renal disease (ESRD) on long-term peritoneal dialysis (PD) with extensive intestinal peritoneal calcifications and a rather uncomplicated long follow-up.

## 1. Case Presentation

We present the case of a 55-year-old female with a history of Down's syndrome, vesicoureteric reflux, and recurrent urinary tract infections since age four that led to ESRD at age 30. Her initial dialysis method was continuous ambulatory peritoneal dialysis (CAPD). The initial CAPD regimen consisted of four, two-liter dwell exchanges per day, using only 1.36% dextrose, with a biocompatible solution, buffered by a mixture of lactate and bicarbonate. The patient was a low average transporter and easily achieved adequate ultrafiltration (>1.0 Lit/day). Her CAPD course was complicated by two mild episodes of peritonitis in the first 2 years. The isolated pathogens were coagulase-negative *Staphylococcus* and Streptococcus viridans, and both were successfully and easily treated with intraperitoneal administration of vancomycin and cefuroxime, respectively. The patient's clinical course was otherwise uncomplicated.

**Figure 1:**
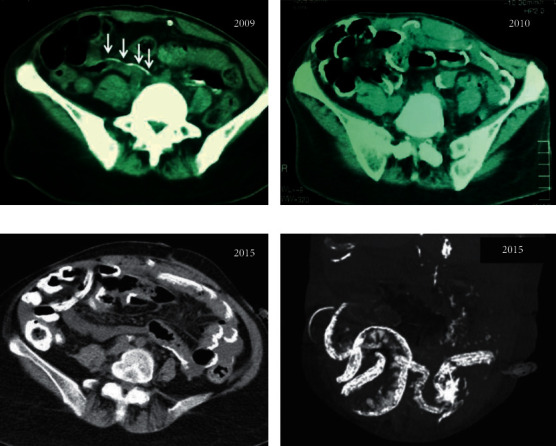
Abdominal CT scans of the patient in 2009 (a), showing mild linear peritoneal calcifications (arrows), which became full-blown calcifications of the visceral small bowel peritoneum a year later in 2010 (b) that remained or minimally increased over the next five years (2015 (c)). (d) Processed sagittal plane image of the 2015 abdominal scan showing the extent of bowel wall calcifications.

Fourteen years following the initiation of PD, she presented with mild abdominal pain and bloody effluent. There were no signs of overt malnutrition or weight loss. She appeared quite well nourished, her blood pressure was 140/70, there was no clinically evident oedema, and hemoglobin values were maintained within normal limits. PD fluid was clear, cell count < 15/mm^3^, and there was no tenderness on examination. Her mother reported regular bowel movements and no fever or nausea. Abdominal computed tomography (CT) (Figure1(a)) showed a—rather expected after long-term PD—focal thickening of the peritoneal membrane suggestive of peritoneal sclerosis but no localization or distension of the bowel loops with no cocooning or signs of bowel obstruction. Inflammation markers (CRP, fibrinogen) were consistently within normal limits. We strongly recommended switching dialysis treatment to hemodialysis, but her mother considered hemodialysis practically impossible in a patient with Down's syndrome and very poor cooperation. She decided to take the risk of continuing PD for as long as possible.

The patient also had a history of secondary hyperparathyroidism. She was treated with alfacalcidol and had a fast and favorable response, with iPTH levels promptly subsiding within a year and being kept within the recommended range (150–350) ever since. Both calcium and phosphate levels, as well as their product, also remained within the recommended range. One year later, she was switched to CCPD after the loss of residual renal function and loss of ultrafiltration due to a progressive change to fast peritoneal transport status.

**Figure 2:**
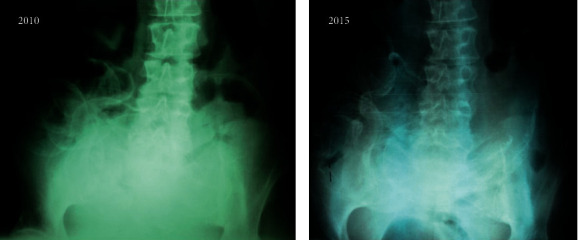
Plain abdominal films of the patient, without any contrast media, showing extensive calcifications of the bowel wall in 2010 (a) and five years later, in 2015 (b).

The patient's clinical course began to deteriorate with symptoms of nausea, loss of appetite, vomiting, and frequent episodes of diffuse abdominal pain. Plain abdominal radiographs revealed extensive linear calcifications along the small intestinal wall, outlining, like a calcified eggshell (Figure 2(a)). An abdominal CT scan (Figure 1(b)) confirmed the diagnosis of calcifying peritonitis, revealing extensive eggshell calcification along the bowel wall, but again no signs of cocooning, localization of the bowel loops, or obstructive ileus were observed. Although there were no formal imaging proofs of encapsulation (EPS), she had the clinical setting and symptoms compatible with sclerosing peritonitis.

The patient and family once again refused the recommended change of dialysis method. Therefore, treatment commenced with tamoxifen 20 mg daily and methylprednisolone 8 mg OD. The patient's symptoms subsided and her clinical condition ameliorated over the next two months. Steroids were tapered to 4 mg every other day over three months, but she was kept on low-dose tamoxifen (10 mg OD) ever since with regular gynecological examination for endometrial hyperplasia every year. Follow-up abdominal CT 5 years later (Figure 1(c)) confirmed the extensive linear calcification of the small intestinal loops, affecting mostly the intestinal splanchnic peritoneum but without any signs of cocooning or intestinal obstruction. Despite the extensive small bowel calcifications, no episodes of bowel obstruction have been recorded till now and the patients' clinical condition remains impressively stable on tamoxifen and methylprednisolone until today.

## 2. Discussion

Peritoneal sclerosis is a rare but well-recognized complication of long-term peritoneal dialysis. Sclerosing peritonitis (SP) usually presents with symptoms of insidious peritoneal (abdominal pain, bloody effluent, and late ultrafiltration failure) and systemic inflammation (loss of appetite, cachexia, malnourishment, low albumin, and rise in CRP). When this leads to the formation of a fibrous cocoon where there is bowel localization and signs and symptoms of intestinal obstruction, the disease is considered encapsulating peritoneal sclerosis (EPS) and eventually leads to the development of full-blown obstructive ileus [1–4]. Pathogenesis of EPS is considered to be multifactorial, and diagnosis is often based on radiological findings of sclerosis, calcification, peritoneal thickening, or encapsulation of the intestines [5].

The incidence of sclerosing peritonitis in peritoneal dialysis patients has been reported to vary in different cohorts between 0.7 and 3.3% but, rather uniformly, in all of the studies, it was clearly associated with the duration of PD [6]. Rigby and Hawley reported a prevalence of confirmed EPS that was reaching 19.4% for patients exceeding 8 years of peritoneal dialysis [4]. Nomoto et al. and Kawanishi et al. also described the major impact of time on PD on the incidence of EPS rising up to more than 17% for those treated for more than 15 years [3, 7].

It has also been suggested that systemic or local inflammation associated with factors such as uremia, malignancies, or peritonitis may be the triggering factor for metastatic peritoneal calcifications. PD-associated peritoneal calcification or calcifying peritonitis (CP) is a rare condition that has been described as an idiopathic syndrome or as part of the clinical manifestations of encapsulating peritoneal sclerosis [8]. It refers to patients with symptoms usually including abdominal pain and bloody or cloudy, sterile effluent that was not associated with formal intestinal obstruction/ileus and had a more benign course than that of “classic” EPS. Only 15 cases have been reported in the literature [8–18]. Several factors have been associated with this rare situation, including frequent peritonitis episodes, calcium-phosphate disturbances, extensive use of vitamin D and calcium products, and secondary hyperparathyroidism. However, due to the small incidence of cases, the causes of this rare condition have not yet been identified and the mechanism underlying the pathogenesis is probably multifactorial.

Clinical manifestations, as well as radiological findings, may differ from the ones seen in EPS. In CP, complete bowel obstruction has not been reported. Radiological findings may include extensive linear calcifications of the parietal and visceral peritoneum, often revealed in a plain abdominal X-ray. However, the patency of the bowel remained intact and no signs of ileus were observed in any of the reported cases. Even more uncommon are the cases in which peritoneal calcification affects mainly the visceral peritoneum. The first report in the literature was from Marichal et al. in 1987 who described two patients of whom one had extensive calcification of the intestinal visceral peritoneum [9]. Both cases were associated with extremely high peritonitis rates (up to one every four months), and the one with visceral peritoneal calcification was much longer on PD (7 years) and had secondary hyperparathyroidism. Francis et al. and Ubara et al. also reported cases of patients with extensive peritoneal calcifications that included the visceral peritoneum of the bowel [10, 18]. Calcifying peritonitis in those cases was also associated with a high number of peritonitis episodes and severe secondary hyperparathyroidism [10].

Our patient had an uncommon complication of PD as she developed extensive calcification of the bowel wall minimally affecting the parietal peritoneum. She did not have a high peritonitis incidence, and although her secondary hyperparathyroidism coincided with the initial presentation of calcification, it was rather promptly and easily controlled. Taking into account the fact that the patient was already in peritoneal dialysis for more than ten years, EPS was considered a possible diagnosis and an evolving process, and so we proceeded in therapy with steroids and tamoxifen, used rather as prophylaxis for a forthcoming progression to full-blown EPS. It seemed like a reasonable approach as rescue therapy, taking as a fact that she and her family declined to change the dialysis modality.

We were pleasantly surprised by her outcome, as it is the first case of CP reported to have acceptable survival without progressing to obstructive EPS or dying, while remaining on PD for more than 9 years since the initial diagnosis, for a total of almost 24 years on PD. A biopsy was not performed, as the patient refused any surgical procedure, so, unfortunately, we cannot support our case with histological evidence of peritoneal calcification.

## Data Availability

All data underlying the results are available as part of the article and no additional source data are required.
